# Risk of subsequent prostate cancer in peptic ulcer patients who received helicobacter pylori eradication therapy: an Asian population-based cohort study

**DOI:** 10.1186/s12894-020-00706-2

**Published:** 2020-08-31

**Authors:** Chu-Wen Fang, Chun-Hao Chen, Chih-Hsin Muo, Shih-Chi Wu

**Affiliations:** 1 Division of Urology, Department of Surgery, Chi Mei Medical Center, Tainan, Taiwan; 2 Management Office for Health Data, China Medical University Hospital, Taichung, Taiwan; 3 School of medicine, China Medical University, Taichung, Taiwan; 4 Trauma and Emergency Center, China Medical University Hospital, No. 2, Yuh-Der Road, Taichung, 404 Taiwan

**Keywords:** *H. pylori*, Prostatic cancer, Peptic ulcer, Cohort study

## Abstract

**Background:**

Studies have shown diverse results regarding the association between *H. pylori* (HP) infection and the risk of malignancy. There is accumulating evidence relating HP infection to urological diseases. We investigated whether there was an association between HP-infected peptic ulcers and the subsequent risk of prostate cancer.

**Methods:**

We collected HP-infected male patients from 1998 to 2008 from the Longitudinal Health Insurance Database (LHID). HP-infected patients were identified as those who had a diagnosis of peptic ulcers upon admission and received HP eradication therapy within 1 year after diagnosis. The date of HP infection diagnosis upon admission was defined as the index date. Patients aged < 20 years or with a cancer history were excluded. For each HP-infected patient, we selected four males without peptic ulcers or a history of HP eradication in the LHID for the comparison cohort according to propensity score matching by age, index year, and comorbidity. The risk of prostate cancer and associated risk factors was assessed by Cox proportional hazard regression.

**Results:**

A total of 2620 HP infection treatment patients and 10,480 matched comparisons were selected. There were 36 patients in the HP-infected treatment cohort and 117 patients in the comparison cohort with documented prostate cancer development (1.52 and 1.21 per 1000 person-years, respectively). Compared to the comparison cohort, the HP infection cohort had a 1.26-fold increased prostate cancer risk in the Cox models after adjusting for matched-pairs (95% CI = 0.87–1.34). There were no significant differences in subsequent prostate cancer development between HP-infected treatment patients and the comparison cohort.

**Conclusion:**

Our findings showed no significant association between HP-infected peptic ulcers and the subsequent risk of prostate cancer. Further studies are warranted to investigate whether this observation is attributable to an HP eradication policy.

## Background

There is a close association between Helicobacter pylori (HP) infection and peptic ulcer disease [1]. HP infection can often be found in most peptic ulcer patients, and eradication of HP has been associated with good outcomes [2]. Thus, medical management has now become the primary choice for the treatment of peptic ulcers [3].

Epidemiologic studies have shown that HP-infected individuals have an increased risk of gastric adenocarcinoma [4, 5] and pancreatic cancer [6], as well as a possible increased risk of colorectal adenocarcinoma [7, 8]. In contrast, studies have shown that HP infection is associated with decreased risks of esophageal adenocarcinoma and esophageal squamous cell carcinoma in Eastern populations. The possible explanation for this association might be attributed to nutritional intake, lifestyle, genetics, tumor biological characteristics and environmental factors [9, 10]. Additionally, an inverse relationship has been shown between HP infection and gastric cardia cancer [11, 12]. These results indicate that HP infection might play different roles in the development of various malignancies.

There is interest in the association between gastrointestinal HP infection and urological diseases, such as benign prostate hyperplasia (BPH) and prostate cancer [13]. One study showed that HP infection should be considered in BPH patients because HP induces apoptosis and demonstrates extragastric effects via the atherosclerotic pathway [14]. A recent study exhibited molecular evidence of the presence of *H. pylori* DNA in prostatic tissue of patients with BPH and prostate cancer [15]. These studies showed that there might be an association between HP infection and urological disease.

We were interested in whether there was an association between gastrointestinal HP infection and urological prostate cancer. Therefore, we performed this population cohort study to evaluate the subsequent risk of prostate cancer in HP-infected peptic ulcer patients.

## Methods

### Study subjects

We collected male patients who received HP infection treatment upon admission from 1998 to 2008 from the Longitudinal Health Insurance Database (LHID). The LHID was a part of the National Health Insurance Research Databases (NHIRD). This database was established by the Taiwan National Health Insurance Administration, Ministry of Health and Welfare. The quality and accuracy of this database has been reported previously [16–18]. There were one million beneficiaries randomly sampled from the year 2000 Registry for Beneficiaries. All Taiwanese residents were obligated to join this program.

**Fig. 1:**
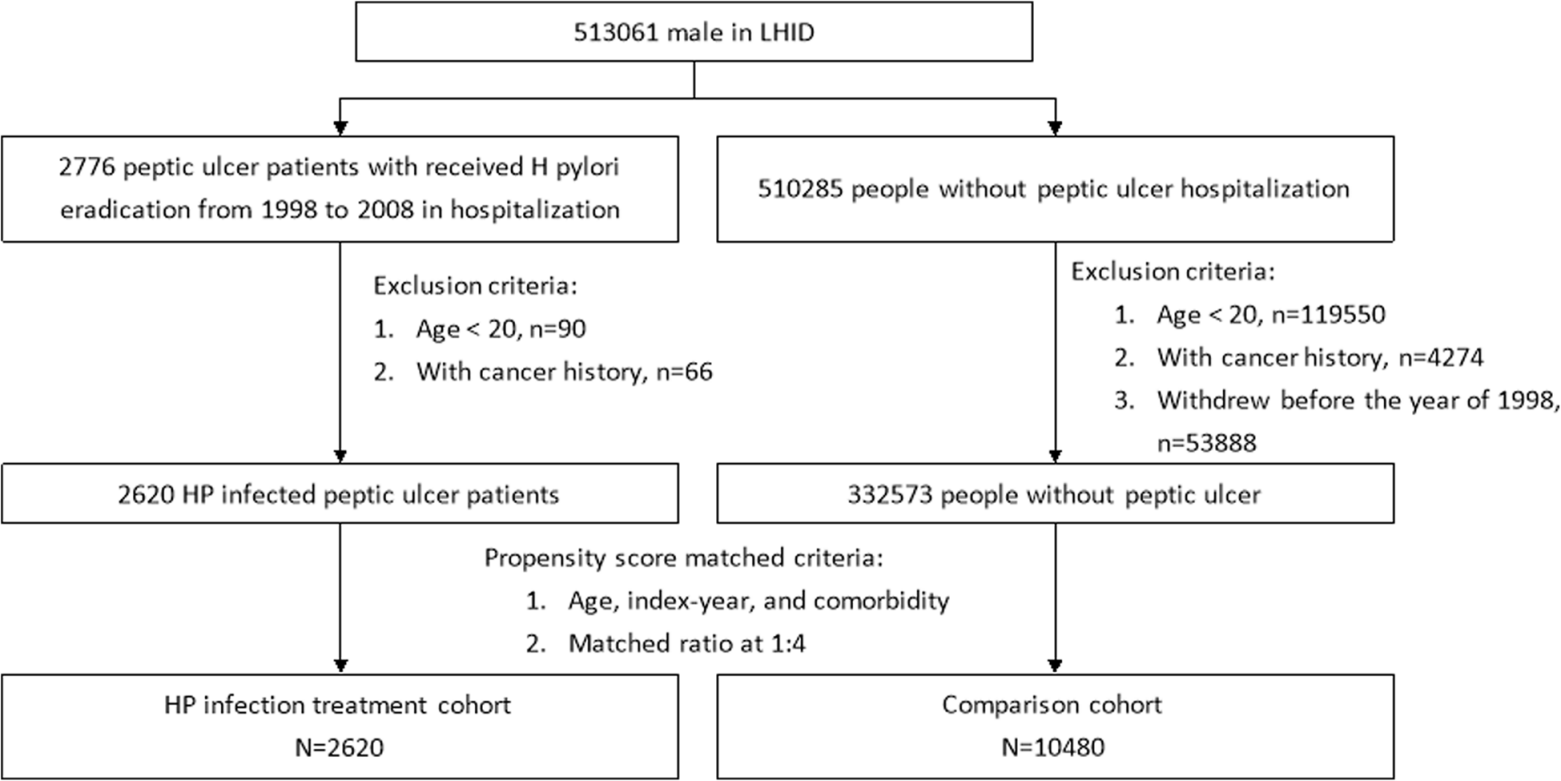
Flow chart of the study population

HP-infected male patients were identified as those who had a diagnosis of peptic ulcer upon admission and who had received HP eradication therapy within 1 year after peptic ulcer diagnosis [The International Classification of Diseases, Ninth Revision, Clinical Modification (ICD-9-CM) 531–533] from 1998 to 2008; these males were defined as the HP infection cohort. The date of HP infection diagnosis upon admission was defined as the index date. In addition, those patients aged < 20 years or with a cancer history were excluded. Because there was a higher than 99% population coverage rate in the Taiwan Bureau of National Health Insurance program, almost all HP-infected peptic ulcer patients received eradication therapy. Thus, it would be difficult to find an HP infection cohort without treatment in the current series. Therefore, for each HP-infected male patient, we selected four males for the comparison cohort who were without prior hospitalization for peptic ulcers or an HP eradication therapy history in the LHID; males in the comparison cohort were selected according to propensity score matching by age, index year, and comorbidity. Because there were no dates of HP infection diagnosis upon admission in the comparison cohort, we randomly assigned a date between 1998 and 2008 as the index date. As a result, there might be HP-infected male patients who visited an outpatient department only and therefore were not hospitalized in the comparison group. However, the exclusion criteria for the comparison cohort were the same as those for the HP infection cohort. A flow chart of the study subjects is depicted in Fig. 1.

This study was approved by the Research Ethics Committee at China Medical University and Hospital. The need for informed consent was waived for all participants [CMUH104-REC2–115]. To comply with research ethics and the Personal Information Protection Act, the identifications of all insured people were shuffled and replaced with surrogate numbers for completion of this research.

### *H. pylori* eradication therapy

HP-infected peptic ulcer patients had received early HP eradication therapy in Taiwan; triple or quadruple therapy included a proton pump inhibitor or an H2 receptor blocker plus clarithromycin or metronidazole and amoxicillin or tetracycline, with or without bismuth. These drug combinations were prescribed within the same prescription order, and the duration of therapy was between 7 and 14 days [19].

### Baseline comorbidity and outcomes

In this study, the following comorbidities were considered (ICD-9-CM): obesity (278), diabetes (250), smoking-related disease (including smoking [305.1], asthma [493], COPD [490–496], ischemic heart disease [410–414], and stroke [430–438]), alcohol-related illness (including alcoholic psychoses [ICD-9-CM code 291], alcohol dependence syndrome [ICD-9-CM code 303], alcohol abuse [ICD-9-CM code 305], alcoholic fatty liver [ICD-9-CM code 571.0], acute alcoholic hepatitis [ICD-9-CM code 571.1], alcoholic cirrhosis [ICD-9-CM code 571.2], alcoholic liver damage [ICD-9-CM code 571.3]), prostatitis (601), BPH (600.0) and sexually transmitted disease (054.1, 091, 096, 098, 099.5).

We considered these comorbidities because there is an increased risk of cancer associated with diabetes [20], alcohol [21, 22] and smoking [23, 24]. Moreover, prostatitis, BPH, and sexually transmitted diseases might play roles in the development of prostate cancer [25, 26]. Therefore, we evaluated these comorbidities in the comparison and HP infection cohorts. These confounders were defined before the index date.

### Diagnosis of prostate cancer

In addition to the ICD-9-CM system, cancer patients were precisely diagnosed by specialists based on pathology and clinical reports; then, they were entered into a registry system of “catastrophic illness patients” in Taiwan. Thus, we identified prostate cancer patients precisely from this “catastrophic illness patients” registry. The “catastrophic illness patients” registry and the LHID were both parts of the NHIRD, and they can be linked to each other by the identification of insurance identification number. In addition, all study subjects were followed from the index date until the development of prostate cancer. Patients without the development of prostate cancer were followed until the date they withdrew from the program or until the end of 2013, whichever came first.

### Statistical analysis

All statistical analyses were performed using SAS software Version 9.4 (SAS Institute Inc., Cary, NC, USA), and the significance level was set at *p* < 0.05; all tests were two-tailed. The number and percentage are presented for categorical variables such as age group (20–49, 50–64 and 65+ years) and baseline comorbidities. The average age is presented as the mean and standard deviation. The difference in age and comorbidity was tested by counting standardized differences between the HP infection and comparison cohorts. The distribution was considered significant between cohorts when the standardized difference was larger than 0.1.

The incidence of prostate cancer was defined as the sum of prostate cancer cases divided by the sum of follow-up years (person-years) in the HP infection treatment and comparison cohorts. Person-years were calculated from the index date until the end point occurred for each study subject. The association between the risk of prostate cancer and the evaluated risk factors was assessed by Cox proportional hazard regression after adjusting for matched pairs. The age- and comorbidity-stratified analyses were also estimated in the HP infection treatment and comparison cohorts using the Cox model as a sensitive test. The cumulative incidence of prostate cancer was plotted using Kaplan-Meier analysis, and the difference between the HP infection treatment and comparison cohorts was assessed with a log-rank test.

## Results

**Fig. 2:**
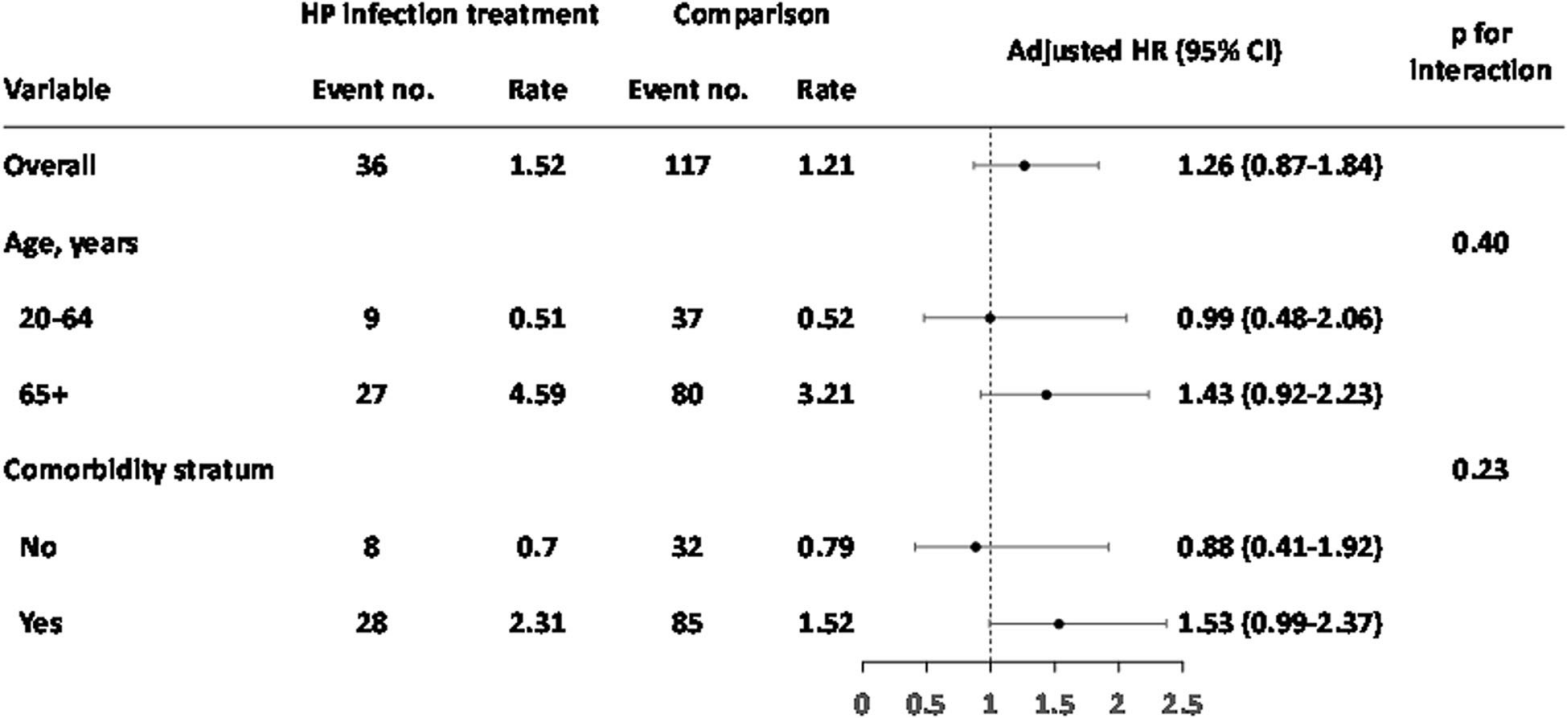
Age- and comorbidity-risk stratified analysis for prostate cancer risk in the HP infection cohort compared with the comparison cohort (adjusted for matched pairs)

**Fig. 3:**
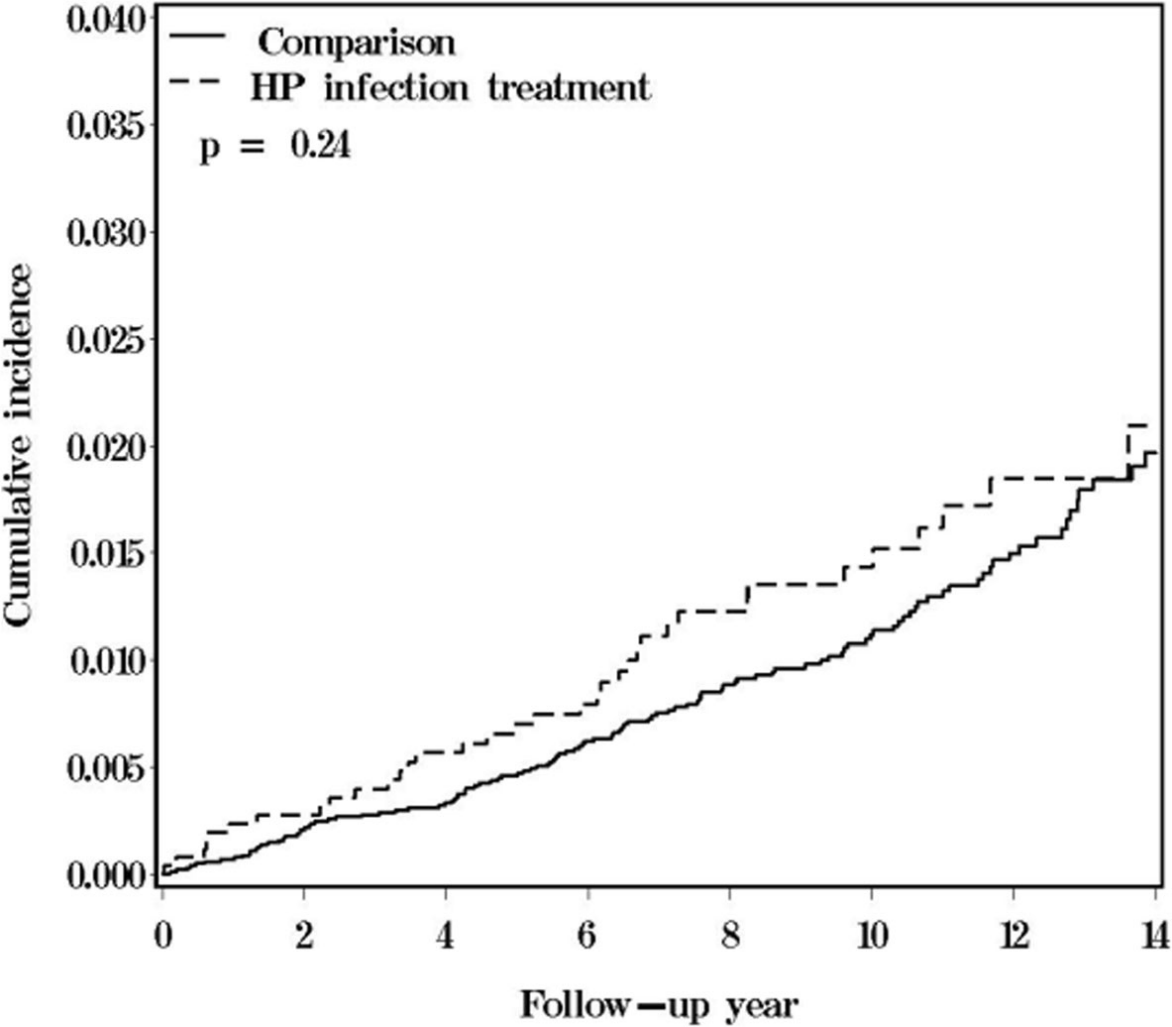
Comparison of cumulative incidence of prostate cancer between the HP infection cohort and comparison cohort

A total of 2620 HP infection treatment patients and 10,480 matched comparisons were selected in this retrospective cohort study (Fig. 1). In the HP infection cohort, the mean age was 54.9 ± 16.5 years (Table 1). There were no significant differences in comorbidity between the two cohorts. In the HP infection cohort, the three most common comorbidities were hypertension (47.3%), diabetes (20.3%), and alcohol-related illness (6.18%). During the follow-up years, there were 36 patients in the HP infection cohort and 117 patients in the comparison cohort who developed prostate cancer; the incidence rates were 1.52 and 1.21 per 1000 person-years, respectively (Fig. 2). The HP infection cohort had a 1.26-fold increased prostate cancer risk compared with the comparison cohort in the Cox models after adjusting for matched pairs (95% CI = 0.87–1.84, Fig. 2). The age- and comorbidity- risk of prostate cancer risk in the HP infection cohort was compared with that in the comparison cohort according to the Cox model. Regardless of age and comorbidity, there were no significant differences in subsequent prostate cancer development between the HP infection treatment and comparison cohorts (Fig. 2). However, after a 14-year follow-up, the cumulative incidence in the HP infection cohort was slightly higher than that in the comparison cohort (2.43% vs. 2.24%) but this difference did not achieve statistical significance (log-rank test, *p* = 0.24, Fig. 3).

**Table 1:** Distribution of age and comorbidity in the HP infection and comparison cohorts after propensity score matching

Variable	HP infection treatment *N* = 2620		Comparison *N* = 10,480		Standardized differences
	n	%	n	%	
Age, years					
20–49	1063	40.6	4386	41.9	0.026
50–64	734	28.0	2731	26.1	0.044
65+	823	31.4	3363	32.1	0.015
Mean (SD)	54.9	(16.5)	54.6	(17.7)	0.020
Comorbidity					
Diabetes	532	20.3	2159	20.6	0.007
Alcohol-related illness	162	6.18	607	5.79	0.016
Smoking-related disease	1238	47.3	5469	52.2	0.099
Prostatitis	37	1.41	235	2.24	0.062
BPH	102	3.89	525	5.1	0.054
Sex transmitted disease	9	0.23	40	0.38	0.028

*SD* Standard deviation

## Discussion

There is interest in HP infection and extragastric diseases. The effects of HP on idiopathic thrombocytopenic purpura, sideropenic anemia, and vitamin B12 deficiency have been well established [27], supporting the notion that some microorganisms can interfere with different biologic processes and cause diseases even remote from the primary infection sites [28].

A hypothetical model for HP infection has been proposed for prostate and bladder diseases [13]. Other studies have demonstrated an association between HP infection and urological diseases [14, 15], and intravesical vaccination against HP infection in chronic cystitis may confer protection against bladder lymphoma [29]. In addition, mucosa-associated lymphoid tissue lymphoma of the urinary bladder disappeared after *H. pylori* eradication therapy [30]. Furthermore, a recent study demonstrated the first molecular evidence of the presence of *H. pylori* DNA in prostatic tissue of patients with BPH and prostate cancer. However, this study was not able to show an association between *H. pylori* infection and prostate cancer due to the limited number of cases [15].

Inflammation may play an important role in the formation of cancer [31, 32], whereas ameliorating inflammation status might reduce the risk of cancer [33]. In addition, evidence has demonstrated an association between inflammation and prostate cancer [34], and HP infection was reported to be associated with chronic prostatitis [35]. In contrast, intriguing studies have demonstrated an inverse relationship between HP infection and gastric cardia cancer [11, 12]. This inconsistency may be explained by the advancement in hygiene and widespread antibiotic use and is supported by the corresponding decrease in HP infection [36].

There were no significant risks of subsequent prostate cancer in HP-infected peptic ulcer patients who received HP eradication therapy (Fig. 2); additionally, there were no differences in the cumulative incidence of prostate cancer between the comparison cohort and peptic ulcer patients who received HP eradication therapy (log-rank test, *p* = 0.24, Fig. 3). There may have been HP-infected male patients who visited an outpatient department only (and therefore were not hospitalized) in the comparison group. The impact on prostate cancer risk in HP infection patients would not be overestimated under this condition.

In Taiwan, there was a higher than 99% population coverage rate in the Taiwan Bureau of National Health Insurance program, and almost all HP-infected peptic ulcer patients received eradication therapy. The current nonsignificant results might be partly explained by a prompt HP eradication policy in HP-infected peptic ulcer patients within 1 year, which results in the suppression of HP infection-induced prostate inflammation. However, further studies are warranted to investigate the association of untreated HP infection and subsequent prostate cancer.

Little is known about the time period between gastrointestinal HP infection and prostate cancer development. In this study, the time period from the index date to an established prostate cancer diagnosis was 5.57 ± 3.84 and 6.39 ± 3.88 years in the HP infection treatment and comparison cohorts, respectively (*p* = 0.27 data not shown). This result demonstrated no differences in the timing of prostate cancer development between patients with and without HP infection treatment. One hypothesis is that the impacts of gastrointestinal HP infection might be diminished after eradication treatment.

There is a close association between aging and cancer [37, 38]. Age is positively associated with the risk of prostate cancer [39]. In addition, the chance of prostate cancer increases rapidly after age 50, and approximately 60% of prostate cancers occur in men older than 65 [40]. Although not significant in this study, older patients (> 65 years old) had a higher incidence of prostate cancer development (Fig. 2), which is consistent with previous reports.

HP infection has been associated with lymphoma of the bladder [30, 41, 42], whereas the most common type of prostate cancer is adenocarcinoma. In this study, these two types of prostate cancer could not be distinguished because of database limitations. However, the associated impact might be minimal because the number of prostate cancers due to lymphoma is very low.

Body mass index (BMI) is a good index to compare comorbidities for prostate cancer [43, 44]. Because there was a lack of exact BMI values in our database, BMI was unable to be considered as a comorbidity for prostate cancer in this study.

There have been studies that demonstrated the impact of gastrointestinal HP infection on the subsequent risk of prostate cancer [15], and the association of HP infection with both chronic prostatitis [35] and an increased risk of prostate inflammation [45]. To our knowledge, there have been no similar epidemiological studies focusing on the impact of eradicated HP infection and subsequent risk of prostate cancer. In this study, no significant associations were noted between HP-infected peptic ulcer patients and the subsequent risk of prostate cancer. HP infection was reported to be associated with chronic prostatitis [35] and might increase the risk of prostate inflammation [45]. Further investigations are needed to elucidate whether our result can be attributed to a prompt HP eradication policy. In addition, further studies might be focused on whether suppression of HP infection has a benefit in reducing HP-induced prostate inflammation or prostate cancer risk.

## Limitations of the study

With reliable diagnosis and a high follow-up rate, our study was strengthened by available data in a large population for longitudinal assessment. However, there were certain limitations. First, lifestyle variables such as drinking, smoking, dietary habits, socioeconomic status, and genetic information were not available for the adjustment of prostate cancer risk estimates. Second, all data used were anonymous, and there was a lack of relevant clinical variables, such as pathology findings, imaging results and laboratory data. Third, the lack of the exact duration between HP infection and HP eradication therapy may have partially neutralized the study findings. Fourth, patients with minor peptic ulcer symptoms may have sought over-the-counter remedies. Therefore, peptic ulcer patients seek for medical services might be a biased group. Additionally, HP infection patients were diagnosed according to codes and those who underwent eradication therapy rather than precise tests. Fifth, there was a lack of data on prostate cancer screening, staging, and status, as well as an established result of HP eradication therapy. Finally, biases related to the retrospective nature of the study and the limited number of patients should be noted.

## Conclusion

In this long-term cohort study, our findings showed no significant association between HP-infected peptic ulcers and the subsequent risk of prostate cancer.

Further studies are warranted to investigate whether this is attributable to the HP eradication policy.

## Data Availability

This study used inpatient claims data from the Taiwan National Health Insurance Research Database (NHIRD). This database contains detailed medical histories of the hospitalized enrollees in Taiwan. Based on the guideline of Taiwan Ministry of Health and Welfare (TMHW), only citizens of the Taiwan are eligible to apply the NHIRD for research projects (https://nhird.nhri.org.tw/en/Data_Protection.html). The database we applied is only limited to our research purpose. All applicants must follow the Computer-Processed Personal Data Protection Law and related regulations of National Health Insurance Administration and NHRI (http://www.winklerpartners.com/?p=987). The ownership of NHIRD is belong to TMHW and the right to use is belong to the researchers. However, other researchers are able to request data access following the regulations of TMHW.

## Abbreviations

HP: Helicobacter pylori
LHID: Longitudinal Health Insurance Database
BPH: Benign prostate hyperplasia
ICD-9-CM: International Classification of Diseases, Ninth Revision, Clinical Modification
